# A Study on the Reuse of Plastic Concrete Using Extended Set-Retarding Admixtures

**DOI:** 10.6028/jres.100.043

**Published:** 1995

**Authors:** Colin Lobo, William F. Guthrie, Raghu Kacker

**Affiliations:** National Ready Mixed Concrete Association, 900 Spring Street, Silver Spring, MD 20910; National Institute of Standards and Technology, Gaithersburg, MD 20899-0001

**Keywords:** drying shrinkage, environmental issues, extended set-retarders, ready mixed concrete, recycling, returned plastic concrete, setting time, stabilizing admixtures, strength, wash water

## Abstract

The disposal of ready mixed concrete truck wash water and returned plastic concrete is a growing concern for the ready mixed concrete industry. Recently, extended set-retarding admixtures, or stabilizers, which slow or stop the hydration of portland cement have been introduced to the market. Treating truck wash-water or returned plastic concrete with stabilizing admixtures delays its setting and hardening, thereby facilitating the incorporation of these typically wasted materials in subsequent concrete batches. In a statistically designed experiment, the properties of blended concrete containing stabilized plastic concrete were evaluated. The variables in the study included (1) concrete age when stabilized, (2) stabilizer dosage, (3) holding period of the treated (stabilized) concrete prior to blending with fresh ingredients, and (4) amount of treated concrete in the blended batch. The setting time, strength, and drying shrinkage of the blended concretes were evaluated.

For the conditions tested, batching 5 % treated concrete with fresh material did not have a significant effect on the setting time, strength, or drying shrinkage of the resulting blended concrete. Batching 50 % treated concrete with fresh materials had a significant effect on the setting characteristics of the blended cocnrete, which in turn affected the water demand to maintain slump. The data suggests that for a known set of conditions, the stabilizer dosage can be optimized within a relatively narrow range to produce desired setting characteristics. The strength and drying shrinkage of the blended concretes were essentially a function of the water content at different sampling ages and the relationship followed the general trend of control concrete.

## 1. Introduction

In a ready mixed concrete plant, the waste water from several processes, including truck-mixer wash water, needs to meet certain criteria, established by environmental agencies, prior to discharge into a storm water drainage system or public waterway. In a plastic state, concrete is a perishable product and disposal of unused concrete left over from a job provides its own set of challenges. Increased environmental regulation requires industry to implement practices that will effectively reduce the quantity of byproduct material requiring disposal.

A survey by the National Ready Mixed Concrete Association (NRMCA) [1] indicated that a majority of concrete producers used a system of settling basins to remove suspended solids from process waste water. Clarified wash water was either discharged into a drainage system, or reused in the plant as batch water in concrete. When returned concrete could not be used, it was either diverted to mechanical reclaiming units or settling basins. In some cases, waste concrete was dumped on the ground and broken up for disposal after it had hardened. Waste hardened concrete and sufficiently drained solid sludge from settling basins was either used as fill material or disposed in landfills or other disposal sites. Increased regulations on the quality of effluent from plant processes and procedures used for solid waste disposal have significantly increased the cost of these disposal techniques.

More recently, Environment Canada published the Environmental Code of Practice for the ready mixed concrete industry in British Columbia [2]. Besides outlining environmental concerns about effluent from ready mix concrete facilities, an overview of current operating practices is presented. The available options for management of waste water and solid waste are outlined. Items discussed in this document are applicable to the ready mixed concrete industry in the United States.

Practices used for handling and disposal of truck wash water vary with job conditions, such as, restrictions on disposal at the construction site, space constraints at the plant, daily production amounts, process control, job schedules, concrete specifications, and local environmental regulations. The Specification for Ready Mixed Concrete, ASTM C 94, permits the use of water from mixer washout operations as mix water in subsequent batches. C 94 places certain criteria on the quality of wash water that can be used as concrete mix water. However, the reuse of wash water may not always be feasible, in which case it has to be disposed in an appropriate manner. Unused returned concrete can be routed to smaller jobs, when available, or used to make pre-cast concrete products at the plant. However, concrete that is past a certain stage in the hydration process will need to be disposed. Mechanical aggregate reclaimers are used to separate aggregates from the returned concrete. The cement slurry water is separated either for disposal or diluted for reuse as mixing water. While initial capital and operating costs of reclaiming units may be high for some of the smaller producers, cost savings from reduced need for settling basins and solids disposal can be significant.

In some cases, blending returned plastic concrete with fresh materials may be a feasible alternative to disposal. Recent technological advances have introduced a chemical admixture [3,4] that can be used to reduce or possibly eliminate the disposal of wash water and returned concrete. The admixture suspends the hydration reaction of cement compounds and allows the holding of plastic concrete or truck-mixer wash water for several hours. The treated concrete or wash water can then be incorporated in the next load of concrete. The admixture is called an extended set-retarding admixture. Some of the commercial products are available as a two component system: a “stabilizer” component, which is the extended set-retarder that slows or stops the hydration of cement grains; and an “activator” component which may be used as an antidote to counteract the effect of the stabilizer and allow cement hydration to continue. The activator component is essentially an accelerating admixture and is used prior to batching fresh material with treated wash water or plastic concrete. In this report, the term “blended concrete” is used for a mixture of stabilized or treated plastic concrete with fresh concrete ingredients. The economic benefits of this process are realized in material savings and in reduction of the costs associated with disposal of byproduct material and plant effluent. However, use of these chemicals requires adequate training of plant personnel to obtain reliable performance. It has been reported that these admixtures do not adversely affect strength and durability-related properties of concrete [5].

This study evaluates some of the processing conditions and the resulting properties of blended concrete containing plastic concrete treated with extended set-retarding admixtures. It should be noted at the outset that the response of a cement and admixture combination is relatively unique. Hence, the conclusions are specific, to a certain extent, to the brands of cement and admixture, as well as the operating conditions, used in this study. However, the results of this study should be qualitatively applicable. The nature and size of this experiment does not allow the determination of well founded uncertainty statements on the (interim) results obtained. Nonetheless the experimental results are valuable as relative, qualitative information for further study.

## 2. Design of the Experiment

### 2.1 Factors in the Experiment

In addition to the normal process control factors that affect the uniform production of concrete, there are several additional processing factors that need to be considered when stabilized concrete is mixed with fresh materials to produce a blended concrete batch. Since it was not feasible to study all of these factors, the first step in planning the experiment was to identify the most important processing factors. The following factors were chosen as variables in this study:

1. the age of concrete when the admixture was added —stabilizer addition time (SAT)
2. the stabilizer dose (SD) used to keep the treated concrete from setting for the desired period
3. the duration for which the stabilized concrete was held prior to the addition of fresh material—concrete addition time (CAT), and
4. the percent (by mass) of treated concrete (PTC) in the blended batch.

**Fig. 1:**
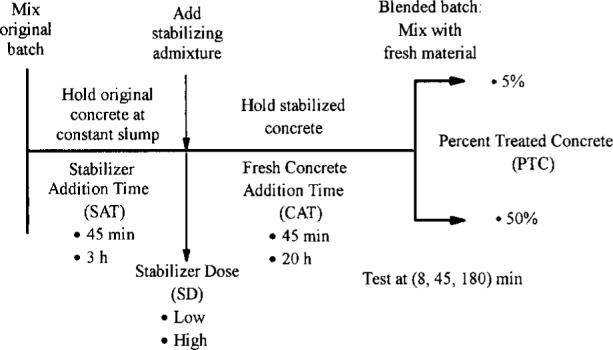
Experimental scheme and factor settings.

A schematic representation of the sequence of operational steps and the related factor settings is presented in Fig. 1.

### 2.2 Factor Levels

Two levels of each factor were chosen to represent practical recycling scenarios of interest. The two levels are referred to as the “low” and “high” levels of the factor. The two settings for each factor are indicated in Fig. 1 and summarized in Table 1.

The low and high settings of the stabilizer addition time, SAT, were set at 45 min and 180 min. The low level, 45 min, represents the earliest time a producer might decide to use a stabilizer. The high level, 180 min, was selected to represent the latest time this particular concrete mixture could be stabilized, from a practical point of view. At 21 °C (70 °F), stabilizing the concrete after 180 min would have required excessive admixture and water addition.

The low and high levels for the stabilizer dosage, SD, were selected so as to hold the treated concrete in a plastic state for the required period. The dosage was estimated based on preliminary tests, described in Sec. 3.2. The selected stabilizer dosage depended on the age of concrete at the time it was treated (SAT), and the duration for which the concrete had to be held prior to batching fresh material (CAT). In this sense these three factors were not mutually independent.

The next processing factor was the percent of treated concrete, PTC, in a blended concrete batch. The levels of this factor were chosen to represent the cases of reuse of wash water and returned concrete, respectively. The mortar fraction, or “butter”, that sticks to the walls of a concrete truck mixer will generally constitute about 1 % of a full load of concrete, or about 270 kg (600 lb) of cement, fine aggregate, and water. The low level of the factor, PTC, was set at 5 % to represent a situation of recycling truck wash water. The high level of PTC was set at 50 % to represent an upper practical limit of recycling returned concrete.

The last factor was the duration of holding treated concrete prior to adding fresh materials, or concrete addition time, CAT. The low level for this factor, 45 min, was chosen to represent recycling wash water or plastic concrete on the same day. The high level, 20 h, was chosen to test the case when stabilized concrete would be held in a truck overnight and batched with fresh materials the next day.

It should be noted that the factor levels used for this experiment were chosen for their research significance and some of them may not be appropriate in ready mixed concrete production operations. For instance, batching fresh materials in a mixer containing 90 min old butter is regularly done and does not typically need the use of a stabilizer. Also, holding treated concrete overnight and combining it with 50 % fresh material is not recommended by the admixture suppliers. Without evidence that the use of recycling conditions more extreme than those recommended by the manufacturer gives satisfactory results, it would be premature to use those levels in production situations.

### 2.3 Constant Factors

The following factors, which have significant effects on the properties of the concrete, were held constant:

1. the concrete temperature, which was maintained at (21±2) °C [(70±3) °F],
2. the concrete ingredients, including cement and admixture brands and aggregates,
3. the concrete mixture proportions for the original and the blended batches, and
4. the concrete slump, which was held relatively constant by retempering as required.

### 2.4 Response Variables

The response variables evaluated in this study were:

1. the setting time of the blended concrete,
2. the compressive strength at 28 d, and
3. the drying shrinkage of 28 d moist-cured specimens after 91 d in air.

### 2.5 Factor Combinations

As described above, four factors, each at two levels, were chosen for the experiment. It is usually possible to ensure that all effects of interest in a designed experiment can be estimated separately from extraneous effects, like batch-to-batch effects. However, in this experiment, it was not possible to do so, due to physical constraints on the operational procedures. Constraints included maintenance of minimum and maximum batch sizes in the available mixers, scheduling tests while the concrete was fresh, and the ability to hold fixed factors at the given levels over time.

As a result of these constraints, operational sequences were scheduled so that 16 “batches” of concrete for different stabilization conditions (all possible combinations of four factors at two levels each) were made from four initial or original batches of concrete, each mixed on a different day. The 16 different stabilization conditions were divided into 4 groups, each of which was made from one of the four original batches. The groups of stabilization conditions to be derived from each original batch were carefully defined to minimize confounding of important effects with the (expected) batch-to-batch variability of the original batches. Nonetheless, the design did not allow for separation of the batch-to-batch effect from the effect of Concrete Addition Time (CAT). The experimental layout, as run,^1^ is given in Table 2. Eight stabilization conditions are summarized in Table 3 with reference to the data presented in Figs. 4, 5, and 6. Each of these conditions tested the percent treated concrete, PTC, in the blended concrete at two levels—5 % and 50 %.

To (partially) resolve the ambiguity arising from the use of this design, it was planned to assess batch-to-batch variability using samples from the original batches. Provided a low batch-to-batch variability of the original concrete was attainable, the effect of CAT on resulting properties of the blended concrete could be estimated. The test results for both the blended and original concretes are discussed in Sec. 4.

## 3. Experimental Procedure

### 3.1 Materials

An ASTM Type I portland cement was used. The composition of the cement is given in Table 4. Natural quartz gravel and a natural sand were used as the coarse and fine aggregates, respectively. Aggregate characteristics are given in Table 5. The coarse aggregate was batched dry and the fine aggregate was batched wet. Added water was adjusted for the aggregate moisture content. A commercially available extended set-retarding admixture was used in the study. Instead of using the “activator” component, a commercially available calcium chloride accelerator, in flake form, was used. Calcium chloride was only used for the batches recycled on the next day, that is, when the concrete addition time was 20 h. Using calcium chloride resulted in some unexpected effects, which are discussed later.

### 3.2 Preliminary Tests

**Fig. 2:**
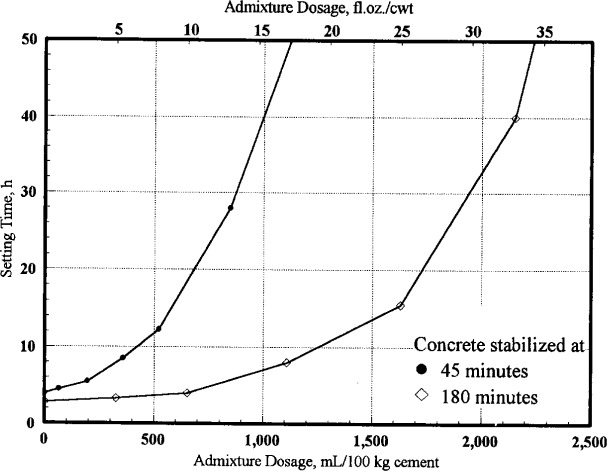
Dosage—set time curves for stabilized concrete.

In order to predict the admixture dosage required to hold concrete without setting, the admixture had to be “calibrated” with the cement. A 0.07 m^3^ (2.5 ft^3^) batch of concrete was mixed and split into four portions. Each portion was held, with mixing and retempering at regular intervals, until the desired ages of 8 min, 45 min, 90 min, and 180 min. At each age, incremental doses of the stabilizing admixture were mixed into the concrete batch and several set time samples, each containing different amounts of admixture, were obtained. The procedure for measuring the setting time is described in Sec. 3.4.1. This data provided dosage versus set time curves for concrete treated at different ages. The dosage versus set time curves for concrete treated at 45 min and 180 min are plotted in Fig. 2. This data was used to estimate the low and high stabilizer doses (SD), based on the required holding time for the stabilized concrete. Figure 2 illustrates that for the same holding time, concrete that was 180 min old when treated required a much higher stabilizer dose than that for concrete that was 45 min old. This set of curves is only valid for this set of materials and concrete temperature of 21 °C (70 °F). While these curves were developed from 100 mm× 100 mm (4 in×4 in) mortar sample, it should be recognized that the setting characteristics of a larger mass of concrete in a laboratory or truck mixer could be significantly different due to differences in temperature history and the rate of heat build up. Continued agitation of stabilized concrete in a truck mixer will also modify the dosage requirements. To be safe, it is advisable to use a stabilizer dose that is somewhat greater than that measured on the mortar sample.

### 3.3 Experimental Sequence

The design mixture proportions of the concrete batches are given in Table 6. Quantities of fresh materials added to treated concrete were determined such that the resulting blended concrete batch would have a similar composition as the original concrete. Batched quantities were corrected for mixer holdback of the mortar fraction based on previously determined factors. Four rotating drum laboratory mixers were used for the sequence of operations.

Mixture ingredients required to produce about 180 kg (400 lb) of concrete were batched and mixed in mixer 1. At 45 min, approximately 55 kg (120 lb) of concrete was transferred to mixer 2 and stabilized with the admixture. The remaining concrete in mixer 1 was held until the concrete was 180 min old at which point it was stabilized with the appropriate admixture dose. Fresh material was batched with the treated concrete at either 45 min or 20 h (CAT) after the stabilizer was added. In the case of recycling on the same day (45 min), the concrete was retained in the mixer which was covered with a polyethylene sheet to prevent evaporation. Concrete that was treated to be recycled the next day was transferred to a metal container and covered with a polyethylene sheet for storage overnight.

At the time of adding fresh material, the stabilized concrete was split into two portions to represent 5 % and 50 % of a blended batch. Fresh materials were batched with the stabilized concrete in mixers 3 and 4 to produce approximately 90 kg (200 lb) of blended concrete. Each original batch thus produced four batches of blended concrete.

Samples taken at 8 min, 45 min, and 180 min, from the four original concrete batches were tested to serve as a benchmark or control measurements for the results of the blended concrete.

Concrete that is stabilized for the purpose of recycling on the next day will be typically over-dosed, to prevent it from setting up in the mixer drum. Prior to batching fresh material, an activator is added to counteract the effect of the stabilizer. In this study, calcium chloride, CaCl_2_, in flake form, was added at the rate of 2 % by total weight of cement in the batch for all overnight blended concrete batches (CAT = 20 h). In order to make appropriate comparisons, calcium chloride was dosed on the basis of the total cement, which included that in the treated concrete and the fresh cement added to the batch. The general practice, however, would be to dose the activator on the basis of the cement content of the stabilized concrete.

In order to have a benchmark or control for concrete recycled overnight, one control batch (a fifth batch) was made with 2 % CaCl_2_, by weight of cement, added to untreated concrete. This batch was tested at the same times using the same protocols used for the other four original batches of concrete.

During the holding time between initial mixing and 180 min, the original and blended batches were mixed at 20 min intervals for 5 min. The concrete was retempered, if required, to maintain approximately a 75 mm (3 in) slump. This operation was based on real time visual evaluation of slump and adjustments of water content. The water additions were recorded and the water content of concrete samples obtained at different ages was calculated from the measured unit weight.

### 3.4 Tests Performed

Samples were obtained from the original and blended concrete batches at ages of 8 min, 45 min, and 180 min. Each series of tests required about 23 kg (50 lb) of concrete. The following tests were conducted:

1. concrete temperature,
2. slump,
3. two 100 mm×200 mm (4 in×8 in) cylinders were molded for a 28 d strength test,
4. one 100 mm×350 mm (4 in×14 in) cylinder was molded with embedded gage studs for drying shrinkage measurements,
5. one 100 mm×100 mm (4 in×4 in) mortar specimen was prepared for measuring the set time, and 6. the 100 mm×200 mm (4 in×8 in) cylinders were weighed to determine the unit weight of fresh concrete.

#### 3.4.1 Set Time

The time of set of concrete was determined by a Proctor penetrometer on the mortar fraction of the concrete sample. Concrete was wet-sieved over a hardware mesh with a 6 mm (1/4 in) opening. Sufficient mortar to fill a 100 mm×100 mm (4 in×4 in) cylinder mold was obtained. Time of set was measured by the penetration resistance of the mortar. Set time is defined as the time elapsed between obtaining the sample and the point when the resistance to penetration was 3.5 MPa (500 psi). Note that this definition of set time is *not* referenced to the time that water touches the cement. The set time, as defined here, is approximately the time it takes for concrete to achieve final set after being placed in its final location. The setting time of the stabilized concrete was also determined and these compared well with the calibration curve shown in Fig. 2.

#### 3.4.2 Unit Weight

Constraints of time and sample quantity prevented the determination of unit weight by ASTM C 138. The 100 mm×200 mm (4 in×8 in) cylinder molds containing fresh concrete were weighed. The volume of the hardened concrete cylinders was determined after they were demolded by weighing them in air and immersed in water. The unit weight was used to calculate the composition of concrete at the various tested ages.

#### 3.4.3 Strength

The 100 mm×200 mm (4 in×8 in) strength cylinders were cured in a moist room and the compressive strength was determined at 28 d according to ASTM C 39. The compressive strength test result is the average of two cylinder breaks.

#### 3.4.4 Drying Shrinkage

The 100 mm×350 mm (4 in×14 in) cylinder specimens were cured in saturated lime water solution for 28 d. The specimens were then dried in air at 21 °C (73 °F) and 50 % relative humidity. Length measurements were obtained at 1 d, 28 d, 56 and 91 d, after the drying was initiated. Drying shrinkage data reported is that of one specimen from each concrete sample.

## 4. Data Analysis and Results

### 4.1 Day-to-Day Variation of Starting Batches

The design chosen for this experiment allowed the different treatments to be run over four days, to accommodate time and equipment constraints, as discussed above. A new batch of concrete was mixed each day to run the four treatments specified in the experimental plan for that day. The first step of the data analysis was a comparison of the four original, untreated batches of concrete to see if there was any evidence of significant batch-to-batch differences. Fresh concrete properties of the four original batches are given in Table 7. Results of 28 d compressive strength tests and 91 d shrinkage in air tests, made on samples taken from the original batches, are given in Table 8. Batch OC is the one batch with calcium chloride prepared as a control for comparing results of the blended batches containing calcium chloride. The tables include results from samples obtained at ages of 8 min, 45 min, and 180 min.

Table 7 indicates that the fresh concrete properties of the original batches at all ages were similar and within the limits of reproducing similar batches of concrete. This is reflected in the strength and shrinkage results in Table 8. The average coefficient of variation of the 28 d strength of the four original batches was 2.1 %. The maximum between-batch standard deviation was for the 8 min samples at about 0.76 MPa (110 psi). This is significantly less than the single laboratory standard deviation for 7 d strength^3^ indicated in precision statement of ASTM C 192, which is 1.40 MPa (203 psi). The between batch variability of the shrinkage results was relatively low.

In order to make appropriate conclusions on the results of the blended batches of concrete, it was important that the between batch variability of the original batches was low, so that these differences could be ignored in the subsequent analysis. Additionally, the data in the tables indicate that the properties of the original batches of concrete were similar at 45 min and 180 min, at which time the original concrete batches were stabilized.

**Fig. 3:**
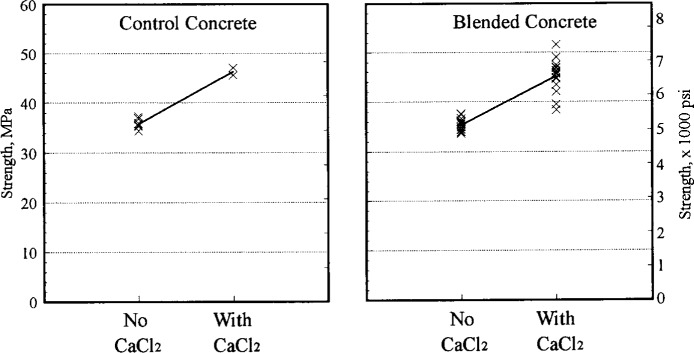
Effect of calcium chloride on concrete strength (8 min samples).

CaCl_2_ had an unexpected effect of the concrete properties. The effect of calcium chloride on properties of the original concrete can be seen in Tables 7 and 8 and Fig. 3. Addition of 2 % calcium chloride, by weight of cement, resulted in reducing the setting time by 1.5 h, increasing the strength by 28 % and increasing the shrinkage by 47 %, for the 8 min samples. Figure 3 also illustrates that on average, concrete strength of the blended concrete was also increased by the same order of magnitude. This effect of CaCl_2_ was not expected and may be unique to the brands of cement and calcium chloride used in this study.

Results for setting time, strength, and shrinkage of the four original batches were averaged to represent the control responses for blended concrete batches representing the same day recycling of treated concrete (CAT = 45 min). Because of the strong effect of calcium chloride of concrete properties, results from batch OC were used as the control responses for blended batches representing the next day recycling of treated concrete (CAT = 20 h).

### 4.2 Main Effects of the Processing Factors

**Fig. 4:**
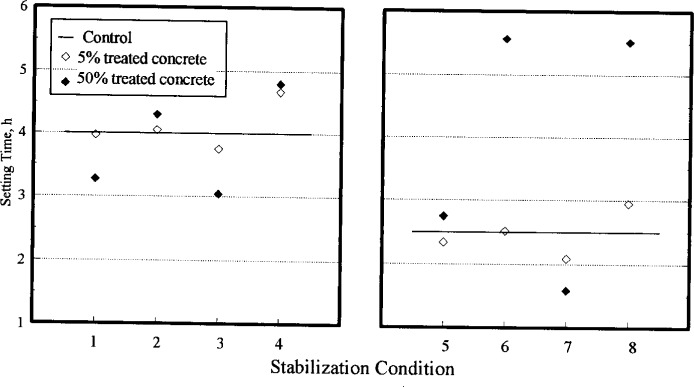
Setting time of blended concrete (8 min samples). Stabilization conditions summarized in Table 3. Plot on left shows data for SAT = 45 min (same day). Plot on right shows data for SAT = 20 h (next day).

**Fig. 5:**
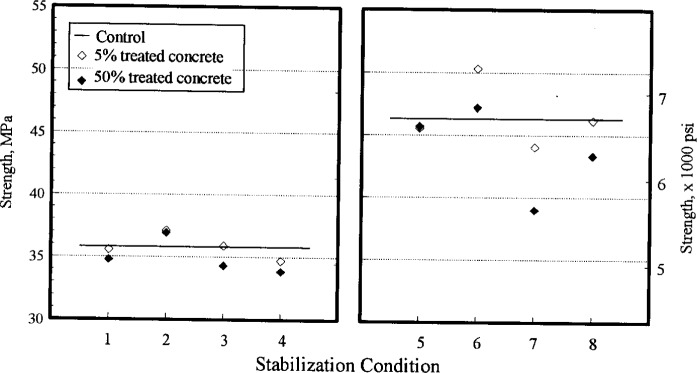
Compressive strength of blended concrete. Stabilization conditions summarized in Table 3. Plot on left shows data for SAT = 45 min (same day). Plot on right shows data for SAT = 20 h (next day).

**Fig. 6:**
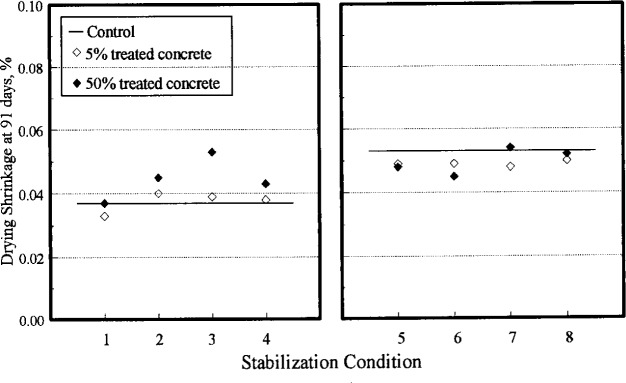
Drying shrinkage of blended concrete. Stabilization conditions summarized in Table 3. Plot on left shows data for SAT = 45 min (same day). Plot on right shows data for SAT = 20 h (next day).

Data for setting time, strength, and drying shrinkage for the blended batches and the corresponding control batches sampled at 8 min are plotted in Figs. 4, 5, and 6, respectively. The stabilization conditions are summarized in Table 3. Comparisons are made for the 8 min samples as the water content of all the samples was essentially similar. Tests made from samples obtained at 45 min and 180 min had varying water contents and these results are addressed later.

The data are divided into two sets—the same day recycling case on the left, and the next day on the right—due to the effect of the unusual CaCl_2_ effect on concrete properties. The data are paired according to low and high dosage levels for concrete stabilized at the same time, and subsequently blended with fresh materials at the same age. The hollow symbol represents blended concrete containing 5 % treated concrete, and the filled symbol is that for 50 % treated concrete.

#### 4.2.1 Setting Time

Figure 4 illustrates the effects of the different stabilization conditions on setting time of blended concrete for the 8 min samples.

In most cases, selecting admixture doses from the “calibration curve,” in Fig. 2, produced set times of blended concrete reasonably similar to those of control. Note that the calibration curve was developed to determine approximate required dosages to hold treated concrete without setting. It was not used to predict setting time of blended concrete.

In one case (condition 7), the dosage used was too low to hold concrete in a plastic state for the required period. About 45 kg (100 lb) of concrete was stabilized at 180 min for the purpose of holding it for at least 20 h. The admixture was dosed based on our best estimate of the cement content of that concrete. The calibration curve in Fig. 2, indicated that this dosage was sufficient to hold concrete for about 28 h to 30 h. However, the concrete had considerably stiffened at 20 h, indicating that setting time determined from a 100 mm×100 mm (4 in×4 in) mortar sample had underestimated of the setting time of the larger mass of concrete.

In Fig. 4, for the same set of recycling conditions, changing the admixture dosage from the low to the high setting resulted in increasing the setting time of the blended batches, as expected. The effect was more pronounced for blended batches containing 50 % treated concrete, again an expected trend.

If fresh ingredients are batched on top of older plastic concrete (without stabilizing admixtures) and mixed, the setting time of the resulting concrete will be accelerated compared to a similar concrete batch not containing the old concrete. Results from a previous study [6] indicated that the older the recycled concrete and the larger the amount in the blended batch, the greater the accelerating effect.

When stabilizers are used, it appears that two opposing mechanisms control setting time: the accelerating effect of old concrete, and the retarding effect of the admixture. For a particular set of stabilizing conditions, the admixture dosage can be optimized to produce a blended batch with the same setting characteristics as that of a control batch. At this optimum dosage, the accelerating and retarding effects should cancel out. If the admixture dosage is less than that optimum amount, the accelerating effect of old concrete is the dominant mechanism. This is seen for the “low” dosage setting in the blended concrete. Increasing from 5 % to 50 % treated concrete in the blended concrete, decreased the setting time further. At the “high” setting of admixture dosage, apparently, the retardation effect of the admixture was dominant, and the setting time of the blended concrete was longer than that of control. Increasing from 5 % to 50 % treated concrete in the blended concrete resulted in an increase in the net admixture in the batch which further retarded the concrete.

If data similar to that in Fig. 4 were developed, it could be used to optimize the admixture dosage for a set of operational conditions to produce blended concrete with the same setting characteristics as a control batch. As seen Fig. 4, a relatively small change in the stabilizer dosage can significantly effect the setting time of blended concrete. Naturally this is easier to do if the specific operating conditions and the routing of a blended concrete is known in advance, which is seldom the case.

It is important to match the setting time of the control, especially if a blended batch of concrete is one of several batches going out to the same job. Not only is this important so that finishers on the receiving end can expect similar characteristics, but as discussed later, the setting characteristics control water addition rates to maintain concrete slump, which in turn controls the properties of the delivered product.

#### 4.2.2 Compressive Strength

The effects of the stabilizing conditions on compressive strength are illustrated in Fig. 5. Strength results of blended batches are compared to control only for the 8 min samples since the water content of the batches at this age were similar, within about 12 kg/m^3^ (20 lb/yd^3^). At the later ages of 45 min and 180 min, the strength of concrete was essentially controlled by the amount of water added to maintain a 75 mm (3 in) slump. The average strength of the control concrete was 35.8 MPa (5186 psi), while that of the one control batch containing CaCl2 was about 46.3 MPa (6715 psi).

For concrete recycled on the same day, the strength of the blended concretes were essentially similar to that of the control concrete. The main effects of each factor, that is the differences between averages at the “low” and “high” settings, can also be estimated. No effect of stabilizer dose was evident. Strength results of concrete stabilized at 180 in (SAT) and then blended averaged 3 % lower than control and 4 % lower than when the stabilizer was added at 45 min. When the recycled batches contained 50 % treated concrete, the average strength was 2 % lower than control. These differences are not of practical significance.

For the concrete recycled on the next day, only condition 7 resulted in significantly lower strength. In this case, the admixture dosage was insufficient and concrete had stiffened considerably when it was mixed with fresh materials. As indicated earlier this set of conditions was extremely severe and is not recommended in practice.

In looking at the main effects for batches containing stabilized concrete blended on the next day, increasing admixture dosage from low to high for the same set of conditions produced slightly higher strengths. Again, this effect was more pronounced for blended concretes containing 50 % treated concrete. The stabilizing admixture disperses cement particles, and this effect is known to produce higher strengths. Concrete that was stabilized at 180 min, on average produced 7 % lower strengths than control and 9 % lower than when the stabilizer was added at 45 min. Strength of blended concrete containing 50 % treated concrete, was 6 % lower than control. While the differences in strength resulting from these conditions may be of practical significance if delivered to the same job, the quality of concrete is still adequate for alternative jobs, if available.

Strength of later age samples was essentially controlled by water addition requirements resulting from a modified setting time. Longer setting times of blended batches resulted in lower water contents and higher strengths, as discussed in Sec. 4.3.

#### 4.2.3 Shrinkage

The shrinkage of 28 d moist cured specimens after 91 d in air is illustrated in Fig. 6. This part of the study was conducted to evaluate the effect of stabilizing admixture in blended concrete on shrinkage characteristics relative to control. This comparison is appropriate for concretes with similar water contents, that is the 8 min samples. The average shrinkage of the four control batches was 0.036 % and that of the one control specimen from batch OC was 0.053 %. Each data point represents the results of one shrinkage specimen at each age.

A point of reference for differences in drying shrinkage data is that for the four original control batches given in Table 8. For the 8 min samples, the coefficient of variation was 4.2 % and the range of results were 8.3 % of the average shrinkage of four batches. Evaluating the main effects for stabilized concrete recycled on the same day, the average shrinkage results of concrete stabilized at 180 min was 21 % larger than that of the control and 15 % larger than concrete batches stabilized at 45 min. Shrinkage of blended concrete containing 50 % treated concrete was on average 24 % more than that of control. However, one batch, condition 3 with 50 % PCT at the high admixture dosage, produced a shrinkage about 47 % larger than control.

For concrete recycled at 20 h, the overwhelming effect of calcium chloride on shrinkage probably clouded any effect of the admixture and stabilization conditions. No significant difference between the recycled batches and the control were evident. The data does not indicate any conclusive effect of stabilizer addition time, stabilizer dose, or percentage of treated concrete in the recycled batch.

At later ages, the shrinkage was controlled by recycling conditions that increased or decreased the setting time with respect to the control batches which in turn determined the water content in the batch at that age, as discussed in Sec. 4.3.

### 4.3 Later Age Properties (45 min and 180 min Samples)

The properties of the concrete as delivered to a job site are of primary interest to both the concrete supplier and purchaser. The job site properties are controlled by the haul time of the concrete, which in turn controls the amount of retempering water required to discharge workable concrete. Concrete temperature, which also has a significant effect on water demand, was held constant at 21 °C (70 °F). A batch plant operator familiar with the materials being used has a general idea of the typical water demand of a mixture and will control the amount of added water to furnish concrete at the required slump to the job. A blended concrete batch might need different amounts of retempering water depending on the stabilization conditions.

**Fig. 7:**
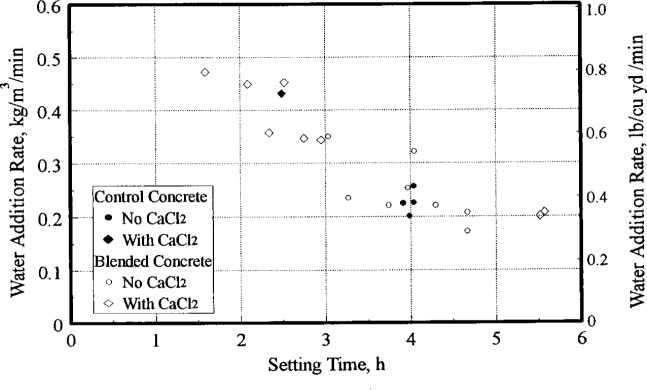
Water demand as affected by setting characteristics.

As indicated earlier, the original and blended concretes were periodically agitated and retempered to maintain a 75 mm (3 in) slump. An estimate of the water addition rate was computed by the difference of the calculated water content of the batch at 180 min and that at initial mixing. The rate of water addition is therefore the amount of water, in kg/m^3^ (lb/yd^3^) per min, required to hold concrete at a 75 mm (3 in) slump for 180 min. This quantity is a rough estimate and is assumed to be linear with time. This rate of water addition is plotted against the setting time of the 8 min sample in Fig. 7. While there is some scatter, the figure illustrates the well known fact that a rapid setting concrete will require a higher rate of water addition to maintain slump. For instance, the control batches of concrete which set at 4 h after initial mixing needed approximately 0.22 kg/m^3^/min (0.4 lb/yd^3^/min) to hold it at a 75 mm (3 in) slump for 180 min. The control concrete batch containing CaCl2 with a setting time of 2.5 h needed approximately double the rate of water addition.

While the relationship in Fig. 7 cannot be used for predictions in practical situations, it serves to illustrate that the properties of the delivered concrete will be a function of the amount of retempering water needed at the job site to discharge concrete at the desired slump, which is a function of the initial setting characteristics of the batch. The set time of blended concrete is controlled by the stabilization conditions or factors. Maintaining an accurate record of water addition will make it possible to ensure that concrete of the desired quality is delivered [7].

**Fig. 8:**
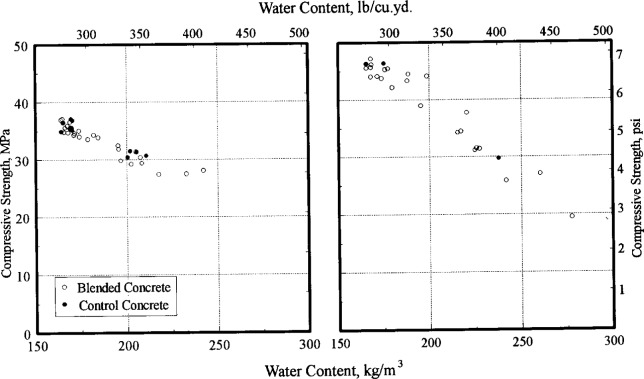
Relationship between water content and strength of concrete. Plot on left shows data for SAT = 45 min (same day). Plot on right shows data for SAT = 20 h (next day).

Figure 8 plots the strength of concrete as a function of the water content. This plot includes measurements on samples obtained at 8 min, 45 min, and 180 min from the control and blended concrete batches. Concrete batches containing calcium chloride are presented in the graph on the right. The data shows the well-known effect that increasing water content results in decreased strength. The data from the control and blended concretes fall within the overall spread of data and no significant separation is evident.

**Fig. 9:**
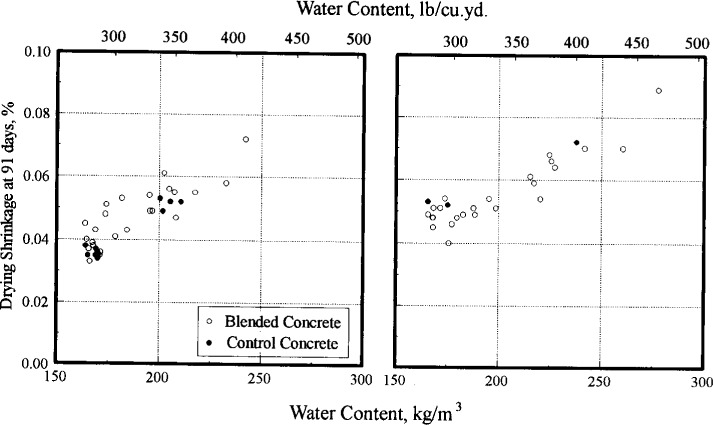
Relationship between water content and drying shrinkage of concrete. Plot on left shows data for SAT = 45 min (same day). Plot on right shows data for SAT = 20 h (next day).

Figure 9 is a similar plot of the shrinkage data. For concrete recycled on the same day, a few points for the blended concretes fall above the general trend of the control batches, indicating a higher shrinkage for the blended batch. These data points represent the more severe conditions of stabilizing concrete at 180 min and recycling at 50 %, as indicated earlier. For concrete recycled at 20 h, the shrinkage of the blended concrete follow the trend of the one control concrete batch, but in most cases, the shrinkage of blended concrete at the same water content was lower than that of the control batch.

In general, the data suggest that at similar water contents, the strength and drying shrinkage of blended concrete is essentially similar to control concrete.

## 5. Conclusions

This study includes only some of the processing factors that may be involved when using extended set-retarding admixtures for the purpose of recycling truck-mixer wash water or returned plastic concrete. Even with this limited scope, it is clear that a significant amount of preliminary testing is necessary to effectively use these admixtures to recycle plastic concrete. However, blended concrete containing stabilized plastic concrete can be used for applications where the setting characteristics are less critical, such as for structural fill or foundation piers. Data representing an upperbound case of recycling stabilized truck-mixer wash water (PCT = 5 %) as batch water in a subsequent concrete batch indicates that the compressive strength and drying shrinkage of the resulting concrete will not be significantly effected.

A calibration curve that determines admixture dosage for different holding times should be developed for a particular cement and admixture combination for various concrete ages and temperatures. Set time indicated by a small mortar sample should be used with caution, especially for long holding times. Setting characteristics of blended concrete were controlled by two opposing mechanisms; the accelerating effect of old concrete and the retarding effect of the admixture. The data suggests that the admixture dosage could be optimized, within a relatively small range, to match the setting characteristics of the control batches.

Comparing concretes at similar water content, more severe recycling conditions, such as stabilizing at 180 min and recycling 50 % treated concrete in a blended batch, resulted in reduced strengths and increased shrinkage. However, at least for compressive strength, these differences were not of practical consequence.

The water demand to maintain slump was related to the modified set time of the blended concrete. Strength and drying shrinkage of blended concrete containing stabilized plastic concrete followed the same relationship with water content as the control concrete.

## Figures and Tables

**Table 1:** Summary of experimental factors

Factor	Setting of factors	
Stabilizer addition time (SAT)	45 min	180 min
Stabilizer dose (SD)	Low	High
Percent treated concrete (PTC)	5 %	50 %
Concrete addition time (CAT)	(45±5) min	(20±2) h

**Table 2:** Layout of experiment, as run

Original batch number	Stabilizer addition time (SAT)	Stabilizer dose (SD)			Concrete addition time (CAT)	Percent treated concrete (PTC)
		Setting	mL/100 kg (fl oz/cwt)			
1	45 min	High	391	(6)	45 min	5 %
	45 min	High	391	(6)	45 min	50 %
	180 min	Low	587	(9)	45 min	5 %
	180 min	Low	587	(9)	45 min	50 %
2	45 min	High	1435	(22)	20 h	5 %
	45 min	High	1435	(22)	20 h	50 %
	180 min	High	2347	(36)	20 h	5 %
	180 min	High	2347	(36)	20 h	50 %
3	45 min	Low	130	(2)	45 min	5 %
	45 min	Low	130	(2)	45 min	50 %
	180 min	High	978	(15)	45 min	5 %
	180 min	High	978	(15)	45 min	50 %
4	45 min	Low	978	(15)	20 h	5 %
	45 min	Low	978	(15)	20 h	50 %
	180 min	Low	1956	(30)	20 h	5 %
	180 min	Low	1956	(30)	20 h	50 %

**Table 3:** Stabilization conditions referenced in Figs. 4, 5, and 6

Stabilization condition	SAT^a^	SD^b^	CAT^c^
1	45 min	Low	45 min (same day) no CaCl_2_
2	45 min	High	
3	180 min	Low	
4	180 min	High	
5	45 min	Low	20 h (next day) with CaCl_2_
6	45 min	High	
7	180 min	Low	
8	180 min	High	

a SAT: Stabilizer addition time.

b SD: Stabilizer dose.

c CAT: Concrete addition time.

**Table 4:** Composition of portland cement, Lot 7324—Series 212D

Composition	Mass fraction, %
CaO	62.5
SiO_2_	20.9
Al_2_O_3_	5.9
Fe_2_O_3_	2.1
SO_3_	3.8
Na_2_O eq.	0.91
MgO	2.8
Potential compound	
(Bogue)	Mass fraction, %
C_3_S	42
C_2_S	28
C_3_A	12
C_4_AF	6

**Table 5:** Characteristics of aggregates

Coarse aggregate, natural quartz gravel, Lot 7290		
Bulk dry specific gravity		2.62
Absorption, %		0.41
Dry-rodded unit weight,	kg/m^3^	1700
	lb/ft^3^	106.2

Sieve		Percent passing
25.0 mm	(1 in)	100
19.0 mm	(3/4 in)	75
12.5 mm	(1/2 in)	50
9.5 mm	(3/8 in)	25
4.75 mm	(No. 4)	0

Fine aggregate, natural sand, Lot 7284		

Bulk dry specific gravity		2.59
Absorption, %		1.16
Fineness modulus		3.12

Sieve		Percent passing
4.75 mm	(No. 4)	100
2.36 mm	(No. 8)	82
1.18 mm	(No. 16)	62
600 μm	(No. 30)	32
300 μm	(No. 50)	9
150 μm	(No. 100)	3
75 μm	(No. 200)	1.5

**Table 6:** Design mixture proportions of concrete batches

Ingredient	Quantity	
	kg/m^3^	lb/yd^3^
Cement	332	560
Water	166	280
Dry fine aggregate	770	1300
Dry coarse aggregate	1070	1800

**Table 7:** Fresh concrete properties of original (control) batches

Property	Original batch	Concrete sampled at					
		8 min		45 min		180 min	
Slump, mm (in)	1	150	(6.00)	75	(3.00)	115	(4.50)
	2	125	(5.00)	70	(2.75)	115	(4.50)
	3	150	(6.00)	75	(3.00)	120	(4.75)
	4	140	(5.50)	75	(3.00)	120	(4.75)
	OC^a^	115	(4.25)	95	(3.75)	105	(4.25)
Temperature, °C (°F)	1	22	(72)	23	(73)	24	(75)
	2	21	(70)	22	(71)	23	(73)
	3	21	(70)	22	(71)	22	(72)
	4	22	(72)	23	(73)	24	(75)
	OC^a^	22	(72)	22	(72)	22	(71)
Set Time, h	1	3.92		3.40		1.83	
	2	4.04		3.35		2.05	
	3	3.99		3.27		NA	
	4	4.04		3.35		2.05	
	OC^a^	2.49		1.73		1.27	
Unit Weight, kg/m^3^ (lb/ft^3^)	1	2379	(148.0)	2387	(149.0)	2365	(147.6)
	2	2379	(148.5)	2393	(149.4)	2371	(148.0)
	3	2387	(149.0)	2392	(149.3)	2368	(147.8)
	4	2382	(148.7)	2397	(149.6)	2379	(148.5)
	OC^a^	2387	(149.0)	2389	(149.1)	2349	(146.6)
Calculated Water Content, kg/m^3^ (lb/yd^3^)	1	164	(277)	166	(279)	200	(338)
	2	169	(285)	170	(287)	205	(346)
	3	170	(286)	170	(286)	202	(340)
	4	169	(285)	170	(287)	211	(355)
	OC^a^	166	(279)	175	(295)	238	(401)

a Batch OC is a control batch with 2 % CaCl_2_ (by weight of cement) added when ingredients were batched.

**Table 8:** Hardened concrete properties of original (control) batches

Property	Original batch	Concrete sampled at					
		8 min		45 min		180 min	
28 day Strength, MPa (psi)	1	34.9	(5060)	36.4	(5283)	30.4	(4408)
	2	35.4	(5140)	35.2	(5105)	31.3	(4535)
	3	37.1	(5378)	35.6	(5158)	31.5	(4563)
	4	35.2	(5165)	36.9	(5345)	30.6	(4443)
(Avg. of 2 cylinders)	Average	35.8	(5186)	36.0	(5223)	30.9	(4487)
	CV	2.6 %		2.1 %		1.6 %	
	OC^a^	46.3	(6715)	46.4	(6728)	29.7	(4308)

91 d Shrinkage in air, %	1	0.038		0.035		0.053	
	2	0.035		0.034		0.052	
	3	0.035		0.036		0.049	
	4	0.037		0.035		0.052	
	Average	0.036		0.035		0.051	
	CV	4.2 %		2.9 %		3.7 %	
	OC^a^	0.053		0.052		0.072	

a Batch OC is a control batch with 2 % CaCl_2_ (by weight of cement) added when ingredients were batched.

## Footnotes

1 As designed, both low and high stabilizer dosages were to be used in each batch, to ensure the ability to separate the SD effect from batch-to-batch variability. However, two high doses were inadvertently used in batch 2 and, correspondingly, two low doses in batch 4. Thus, the ability to estimate the SD effect also depended on low between-batch variability for the original batches.

3 No precision result is listed in the standard for 28 d strength.
